# Job and Employee Satisfaction Among Healthcare Staff in a Tertiary Hospital: A Cross‐Sectional Study

**DOI:** 10.1155/jonm/9983570

**Published:** 2026-06-02

**Authors:** Miroslav Jelic, Aleksandar Ristic, Filip Djokovic, Verica Jovanovic, Ivan Soldatovic, Bojana Salovic, Stefan Mandic-Rajcevic, Milan Perovic

**Affiliations:** ^1^ Department for the Implementation of International Social Security Agreements, National Health Insurance Fund, Belgrade, Serbia; ^2^ Faculty of Medicine, University of Belgrade, Belgrade, Serbia, bg.ac.rs; ^3^ Outpatient Unit, Clinic for Gynaecology and Obstetrics Narodni Front, Belgrade, Serbia; ^4^ Faculty of Health, Legal and Business Studies, Singidunum University, Valjevo, Serbia, singidunum.ac.rs; ^5^ Faculty of Organizational Studies Eduka, Belgrade, Serbia; ^6^ Department for Communicable Disease Prevention and Control, Institute of Public Health of Serbia Dr Milan Jovanovic Batut, Belgrade, Serbia; ^7^ Institute of Medical Statistics and Informatics, Faculty of Medicine, University of Belgrade, Belgrade, Serbia, bg.ac.rs; ^8^ Department of Assisted Reproduction, Clinic for Gynaecology and Obstetrics Narodni Front, Belgrade, Serbia; ^9^ School of Public Health and Health Management, Faculty of Medicine, University of Belgrade, Belgrade, Serbia, bg.ac.rs; ^10^ Institute of Social Medicine, Faculty of Medicine, University of Belgrade, Belgrade, Serbia, bg.ac.rs

**Keywords:** extrinsic and intrinsic factors, healthcare staff, job satisfaction, obstetrics and gynaecology

## Abstract

**Background:**

Job satisfaction significantly influences the quality of patient care. Healthcare staff working with vulnerable patients in tertiary obstetrics and gynaecology hospitals faces specific work challenges and higher expectations from patients. The secondary data analysis from the National Survey of Employee Satisfaction aimed to evaluate job satisfaction and its association with intrinsic and extrinsic factors.

**Methods:**

A cross‐sectional study was conducted involving 385 employees (physicians, nurses, administrative and technical staff) at two tertiary obstetrics and gynaecology hospitals. Data were collected using a self‐administered, anonymous questionnaire (National Survey of Employee Satisfaction). This single instrument assessed demographic characteristics and 14 factors of the work environment and working conditions. Participants rated their level of satisfaction on a 5‐point Likert scale, with scores ranging from 1 (‘very dissatisfied’) to 5 (‘very satisfied’).

**Results:**

Participants were aged 35–54 years, with nurses comprising the largest proportion of the sample (60.3%). The greatest proportion reported dissatisfaction with their salary (61.8%), while the highest level of satisfaction was reported for work equipment (41.5%). Overall job satisfaction varied across professional categories of employees. Exploratory factor analysis among healthcare professionals identified two underlying dimensions of job satisfaction: extrinsic (hospital‐related) and intrinsic (profession‐related) factors, which together explained 59% of the total variance. Both factors were significantly associated with overall job satisfaction, with extrinsic factors showing a slightly stronger correlation.

**Conclusion:**

Improving non‐financial incentives (recognition of work, organizational support and workload management) may enhance healthcare staff engagement, communication, staff morale, retention and service quality. Given the identified intrinsic and extrinsic dimensions of job satisfaction and their significant association with overall job satisfaction, this empirically derived framework may be used for comparative analyses across different healthcare contexts and may inform the development of more targeted measurement tools and intervention models.

## 1. Introduction

Healthcare staff provide direct and indirect care to patients, and their employee and job satisfaction substantially influence efficiency and motivation, thereby improving both the quantitative and qualitative aspects of treatment outcomes [1, 2]. Improved employee and job satisfaction lead to an increase in the quality of provided healthcare and greater satisfaction of healthcare users [3]. The performance of healthcare professionals is inextricably linked to patient safety [4]. Low level of job satisfaction among nurses is a predictor of adverse outcomes and complications during the treatment of postoperative patients [5]. The job satisfaction of healthcare staff and their motivation are linked with the outcomes of patient treatment and influence all healthcare users. Management of healthcare institutions is difficult and complicated task, requiring balance between all parties in the healthcare system: patients on one side and medical and non‐medical staff on the other, considering the needs of the whole community. Economic reasons dictate a reduction in the number of employees, limitation of salaries, while the increase in patient satisfaction is a paramount goal. Therefore, the only factor that can be acted upon to reconcile these opposed requirements is job satisfaction of employees [6, 7].

Tertiary healthcare centres require highly specialized equipment and expertise and are dedicated to specific subspeciality care. They combine hospital services with education and medical research and represent the top of healthcare system pyramid, providing care to the most demanding, seriously ill patients, using multidisciplinary approach, sophisticated diagnostic and therapeutic equipment. Greater expectations from employees in tertiary centres are present among patients [8], especially those in vulnerable populations, such as pregnant women [9]. Those expectations are related not only to healthcare professionals but also to administrative and technical staff, especially regarding plan and schedule of institution functioning, space maintenance and business process management [10]. Simultaneously, employees of tertiary centres face higher job demands, workplace stress, excessive hours of work and inadequate periods of rest and recuperation [11, 12] than those in primary and secondary centres, which results in decreased job satisfaction. Job dissatisfaction was registered in 64.8% employees in tertiary care centres [12].

Job satisfaction factors are conditioned by intrinsic motivation factors (derived from interest and fun or challenge) and extrinsic factors that bring some instrumental value (praise, reward, cooperation and competition with colleagues, salary, security, working conditions, establishing the desired interpersonal relationships with colleagues and superiors) [13]. The importance of internal and external motivation in determining the degree of job satisfaction is emphasized among health professionals. Due to the nature of their work, healthcare professionals must make many sacrifices, and they are ready to make such a sacrifice, guided primarily by internal and much less external motivation, driven by dedication and love for the job and by great empathic potential [14].

Few studies have examined the association of intrinsic and extrinsic motivating factors with job satisfaction, and none have examined this relationship in a sensitive and specific milieu of tertiary obstetrics and gynaecology hospitals. Therefore, we aimed to determine factors that influence the most different aspects of job and employee satisfaction, and overall job and employee satisfaction among all employees and within each professional category in tertiary obstetrics and gynaecology hospitals. The main study objective was to identify intrinsic and extrinsic factors among job and employee satisfaction and to evaluate their correlation with overall job satisfaction.

## 2. Materials and Methods

### 2.1. Study Design

This secondary data analysis used the data from the National Survey of Employee Satisfaction in public healthcare institutions in 2019 conducted by the Institute of Public Health of Serbia ‘Dr Milan Jovanović Batut’. This national survey is based on a questionnaire on employee satisfaction, which was anonymously filled out by voluntary survey participants. The questionnaire used in this study is an instrument developed by the Commission for the Improvement of the Quality of Healthcare of the Ministry of Health of the Republic of Serbia, and this questionnaire evaluates both job and employee satisfaction. The questionnaire was pilot‐tested and validated in 2005 in five healthcare institutions of different types and has been in use since then [15]. Every public healthcare institution in Serbia is obliged by the Institute of Public Health of Serbia ‘Dr Milan Jovanović Batut’ to conduct an annual national survey of employee satisfaction. The Institute provides the survey in paper format to each healthcare institution in sufficient quantity. Hospital managers and department heads are responsible for distributing of questionnaires to all employees, while participation in the survey is entirely voluntary. Employees who choose to participate complete the job satisfaction questionnaire and deposit it in designated collection box. After some time period, the sealed boxes are transferred to the Institute of Public Health of Serbia ‘Dr Milan Jovanović Batut’, where the completed questionnaires are entered into an electric database. Both tertiary obstetrics and gynaecology hospitals in our study conducted the survey in 2019 in accordance with this standardized procedure. For the purpose of our research, we submitted a detailed study protocol to the Ethics Committee of the Institute of Public Health of Serbia ‘Dr Milan Jovanović Batut’ and formally invited its representatives to collaborate. The Institute extracted from its electronic database all survey responses from employees of the two participating clinics for the year 2019. The study was approved by the Ethics Committee of the Institute of Public Health of Serbia ‘Dr Milan Jovanović Batut’ (Decision No. 2271/1 of 16 March 2020).

### 2.2. National Survey of Employee Satisfaction

The first part of the questionnaire was related to the demographic characteristics and general data of the participants, such as gender, age, profession, educational level and having a managerial position. The second part consisted of questions regarding 14 aspects (factors) of the work environment and working conditions that affect job satisfaction: (1) adequacy of work equipment, (2) available time to perform tasks, (3) available time to work with patients, (4) job autonomy, (5) possibility to use all personal knowledge, skills and abilities at work, (6) appreciation and evaluation of work, (7) cooperation with colleagues, (8) cooperation with superiors (managerial staff), (9) patients’ attitude towards employees, (10) possibility of professional development and continuous education, (11) salary, (12) satisfaction with management and organization of work in the institution, (13) clear work instructions and (14) possibility to express ideas to superiors (managerial staff). After the analysis of demographic and general data of healthcare staff, we evaluated the 14 factors of the work environment and working conditions that affect job satisfaction. Four factors were identified as intrinsically motivated (intrinsic factors): (1) available time to perform tasks, (2) available time to work with the patient, (3) relationship with the patient and (4) workplace stress. The remaining 10 factors were identified as extrinsically motivated (extrinsic factors) [13]. The participants responded to these questions on a 5‐point Likert scale: ‘Very dissatisfied’, ‘Dissatisfied’, ‘Neutral’, ‘Satisfied’ and ‘Very satisfied’. In case if the job satisfaction factor could not be applied to the participant, the answer could be ‘Does not apply to me’. Therefore, the minimum possible score per item was 1 and the maximum was 5. Factor analysis was performed on the 14 items, and two components (intrinsic and extrinsic factors) were extracted. Overall job satisfaction was assessed separately using a single‐item measure rated on the same 5‐point Likert scale (range 1–5). Factor analysis was performed on the 14 items, and two components were extracted. Based on the rotated solution, the components were interpreted as extrinsic and intrinsic job satisfaction factors. The extracted components were saved as standardized factor scores (mean = 0) and subsequently used in correlation analyses to examine their association with overall job satisfaction. The National Survey of Employee Satisfaction is provided in full in Appendix A.

### 2.3. The Setting

In Serbia, healthcare services are provided through a network of healthcare institutions. In general, there are three levels of healthcare: primary, secondary and tertiary. Our study analysed data collected at two obstetrics and gynaecology hospitals at the tertiary level of healthcare in Belgrade, the capital city of Republic of Serbia. These two hospitals, the Clinic for Gynaecology and Obstetrics ‘Narodni Front’ and the Clinic for Gynaecology and Obstetrics of the Clinical Centre of Serbia, are the two largest hospitals of this type in Serbia out of a total of six gynaecology and obstetrics hospitals in Serbia. In total, 389 questionnaires were completely answered by the employees.

### 2.4. Statistical Analysis

Descriptive and analytical statistical methods were used in this study. All data were nominal (sociodemographic characteristics and employment status) and ordinal (questionnaire) and presented using count and percent. Analytical statistical methods comprised of Spearman’s correlation analysis and factor analysis. The Spearman correlation analysis was performed to obtain significant correlations between ordinal variables (or at least one ordinal variable in the analysis), while factor analysis was performed to create factors out of 14 questions of the Questionnaire for the Employee Satisfaction. Obtained factors were recorded as separate scores (dimensions) for intrinsically and extrinsically conditioned job satisfaction factors, which were then correlated with the overall job satisfaction of employees. The Kaiser–Meyer–Olkin measure of sampling adequacy (very good > 0.8, > 0.9 excellent) was used to analyse the adequacy of the data in order to identify those factors among the evaluated factors that describe a large percentage of the variability of the whole data set. Bartlett’s test of sphericity was used to analyse the adequacy of the data in terms of excluding significant correlations between the evaluated observation features. The communalities model analysed the percentage of variability contributed by each variable in the model. Correlation between overall job satisfaction and extracted factors was performed in the overall sample and within each profession. Data were processed using IBM SPSS Statistics v29.0 (IBM Corp., Armonk, NY, USA, released in 2022). All *p* values less than 0.05 were considered significant.

## 3. Results

Out of 389 correctly and completely answered questionnaires of employees, we have excluded four healthcare staff, whose profession was not subject to this investigation (one pharmacist and three dentists), from the analysis. Finally, the analysis included 175 questionnaires from the Clinic for Gynaecology and Obstetrics of the Clinical Centre of Serbia and 210 questionnaires completed at the Gynaecology and Obstetrics Clinic ‘Narodni Front’, which represented 61% response rate. The data referred to 69 physicians (69 out of 115, response rate 60%), 232 nurses and medical technicians (232 out of 357, response rate 65%), 29 administrative staff (29 out of 52, response rate 56%), 24 technical staff (24 out of 38, response rate 63%) and health associate professionals (31 out of 51, response rate 61%). Medical technicians include staff performing clinical support roles such as phlebotomists, physician associates and other patient‐care‐related technical tasks. We grouped them with nurses due to their direct involvement in patient care, whereas technical staff have equipment‐ or technology‐focused roles, such as IT or medical device support, roles regarding maintaining appropriate environmental conditions, including ventilation, temperature control and air quality, in operating theatres, laboratories, patient rooms and intensive care units. Moreover, they are responsible for management of utility systems, including medical gases, electrical supply and water systems. They also support sterilization processes and monitor equipment used in infection control.

General characteristics of study participants are shown in Table 1. The largest numbers of participants were female, within the age category of 35–54 years and professional category of nurse/medical technician.

**TABLE 1:** General characteristics of employees (*n* = 385).

	*n* (%)
Gender female	344 (89.4%)
Age	
Less than 35	107 (27.8%)
35–54	208 (54.0%)
55 and above	70 (18.2%)
Profession	
Physician	69 (17.9%)
Nurse/medical technician	232 (60.3%)
Health associate professionals	31 (8.1%)
Administrative worker	29 (7.5%)
Technical worker	24 (6.2%)
Managerial position	55 (14.3%)

The analysis of the perception of working conditions of employees in tertiary obstetrics and gynaecology hospitals in Belgrade is presented in Table 2. The largest percentage of staff were very dissatisfied or dissatisfied with their salary (33.5% and 28.3%), followed by available time for patients (13% and 21.8%). On the other hand, the staff were very satisfied and satisfied with work equipment (8.8% and 32.7%), followed by the available time to perform tasks (7.8% and 29.9%).

**TABLE 2:** Participant’s perceptions towards working conditions (*n* = 385).

		Very dissatisfied	Dissatisfied	Neutral	Satisfied	Very satisfied	Does not apply to me
Work equipment	*n* (%)	32 (8.3)	77 (20.0)	113 (29.4)	126 (32.7)	34 (8.8)	3 (0.8)
Available time to perform tasks	*n* (%)	44 (11.4)	65 (16.9)	131 (34.0)	115 (29.9)	30 (7.8)	0 (0.0)
Available time for patients	*n* (%)	50 (13.0)	84 (21.8)	100 (26.0)	83 (21.6)	22 (5.7)	46 (11.9)
Job autonomy	*n* (%)	47 (12.2)	73 (19.0)	111 (28.8)	101 (26.2)	49 (12.7)	4 (1.0)
Salary	*n* (%)	129 (33.5)	109 (28.3)	88 (22.9)	39 (10.1)	19 (4.9)	1 (0.3)

Absolute numbers and corresponding percentages of employee responses in the five categories of responses related to the relationship with management are presented in Table 3.

**TABLE 3:** Participant’s perceptions towards relationship with supervisors (*n* = 385).

		Very dissatisfied	Dissatisfied	Neutral	Satisfied	Very satisfied
Appreciation and evaluation of work	*n* (%)	88 (22.9)	78 (20.3)	97 (25.2)	87 (22.6)	35 (9.1)
Cooperation with superiors	*n* (%)	38 (9.9)	47 (12.2)	111 (28.8)	122 (31.7)	67 (17.4)
Management and organization of work	*n* (%)	65 (16.9)	81 (21.0)	119 (30.9)	91 (23.6)	29 (7.5)
Clear work instructions	*n* (%)	61 (15.8)	60 (15.6)	117 (30.4)	112 (29.1)	35 (9.1)
Possibility to express ideas to superiors	*n* (%)	62 (16.1)	79 (20.5)	103 (26.8)	106 (27.5)	35 (9.1)

Six percent of participants were very dissatisfied with direct cooperation with colleagues, 10.4% were dissatisfied, 23.4% were neither satisfied nor dissatisfied, 41.8% were satisfied and 18.4% were very satisfied. When asked about the patient’s attitude towards employees, the answer ‘does not apply to me’ was given by 6.8% of respondents and such an answer was given by employees in the administration, technical services. The same question was answered as ‘very dissatisfied’ by 9.6% of respondents, ‘dissatisfied’ by 15.1%, ‘neutral’ by 24.4%, ‘satisfied’ by 30.6% and ‘very satisfied’ by 13.5% of respondents (data shown only as a text not in the table).

Overall, among the 385 participants, the largest proportion reported a neutral level of overall job satisfaction (40.0%), followed by those who were dissatisfied (20.5%) or satisfied (23.6%). Smaller proportions reported being very dissatisfied (10.9%) or very satisfied (4.9%). These data are shown in Table 4.

**TABLE 4:** Overall job satisfaction among participants (*n* = 385).

	*n* (%)
Very dissatisfied	42 (10.9%)
Dissatisfied	79 (20.5%)
Neutral	154 (40.0%)
Satisfied	91 (23.6%)
Very satisfied	19 (4.9%)

Correlation between each job satisfaction factor and overall job satisfaction among different professional categories of employees, as well as the correlation between the overall job satisfaction and each job satisfaction factors among all employees is shown in Table 5.

**TABLE 5:** Correlation of job satisfaction factors with the overall job satisfaction across professional categories (*n* = 385).

	Physician	Nurse	HAP	AW	TW	All
Intrinsic factors						
Workplace stress	−0.25^∗^	−0.420^∗∗^	−0.161	−0.304	−0.482^∗^	−0.345^∗∗^
Available time to perform tasks	0.320^∗∗^	0.462^∗∗^	0.518^∗∗^	0.410^∗^	0.580^∗∗^	0.431^∗∗^
Available time for patients	0.284^∗^	0.474^∗∗^	0.354^a^	0.512^a^	0.061	0.401^∗∗^
Relationship with the patient	0.195	0.381^∗∗^	0.332^a^	0.575^∗^	0.320	0.345^∗∗^
Extrinsic factors						
Job autonomy	0.371^∗∗^	0.411^∗∗^	0.330^a^	0.303	0.301	0.387^∗∗^
Ability to use all knowledge and skills	0.419^∗∗^	0.462^∗∗^	0.338^a^	0.463^∗^	0.537^∗∗^	−0.451^∗∗^
Appreciation and evaluation of work	0.420^∗∗^	0.534^∗∗^	0.488^∗∗^	0.504^∗∗^	0.512^∗^	−0.506^∗∗^
Cooperation with colleagues	0.280^∗^	0.442^∗∗^	0.148	0.256	0.473^∗^	0.369^∗∗^
Cooperation with superiors	0.455^∗∗^	0.511^∗∗^	0.269	0.308	0.427^∗^	0.456^∗∗^
Professional development and education	0.315^∗∗^	0.424^∗∗^	0.459^∗^	0.502^∗∗^	0.266	0.420^∗∗^
Salary	0.224^a^	0.404^∗∗^	0.442^∗^	0.203	0.189	0.356^∗∗^
Management and organization of work	0.458^∗∗^	0.447^∗∗^	0.741^∗∗^	0.478^∗∗^	0.332	0.481^∗∗^
Clear work instructions	0.426^∗∗^	0.530^∗∗^	0.574^∗∗^	0.506^∗∗^	0.132	0.493^∗∗^
Possibility to express ideas to superiors	0.423^∗∗^	0.504^∗∗^	0.375^∗^	0.409^∗^	0.123	0.450^∗∗^
Work equipment	0.337^∗∗^	0.267^∗∗^	0.421^∗^	0.480^∗∗^	0.443^∗^	0.336^∗∗^
Decreased job satisfaction over past 5 years	−0.630^∗∗^	−0.505^∗∗^	−0.527^∗∗^	−0.413^∗^	−0.121	−0.511^∗∗^

*Note:* *p* values were obtained using Spearman’s correlation.

Abbreviations: AW, administrative worker; HAP, health associate professional; TW, technical worker.

^∗^ *p* < 0.05.

^∗∗^ *p* < 0.01.

^a^ *p* < 0.1.

Further analysis was related referred only to healthcare professionals (physicians, nurses and medical technicians). The data available for analysis were adequate, which was confirmed by the *Kaiser–Meyer–Olkin Measure of Sampling Adequacy* (KMO = 0.928), indicating it was possible to extract factors that describe a large percentage of the variability of the whole data set. *Bartlett’s test of Sphericity* also indicated the adequacy of data, and that observed variables were not significantly correlated among themselves (*χ* ^2^ = 2536.709; *p* < 0.001). Analysis of the percentage of variability that each factor ‘gives’ to each variable in the Communalities model is shown in Table 6.

**TABLE 6:** Rotated component matrix^a^ (*n* = 385).

	Component		Extraction
	Extrinsic	Intrinsic	
Work equipment	**0.512**	0.239	0.319
Available time to perform tasks	0.373	**0.707**	0.639
Available time for patients	0.367	**0.711**	0.640
Job autonomy	**0.682**	0.395	0.621
Possibility to work using all knowledge, skills and abilities	**0.741**	0.332	0.659
Appreciation and evaluation of work	**0.726**	0.414	0.699
Cooperation with colleagues	**0.725**	0.204	0.567
Cooperation with superiors	**0.807**	0.283	0.731
Relationship with the patient	0.341	**0.489**	0.356
Professional development and education	**0.713**	0.241	0.567
Salary	0.406	0.521	0.436
Management and organization of work	**0.757**	0.262	0.641
Clear work instructions	**0.748**	0.358	0.687
Possibility to express ideas to superiors	**0.822**	0.193	0.713
Workplace stress (recoded)	0.045	**0.758**	0.576
*Cumulative % variance explained*	*51.9%*	*59.0%*	

*Note:* Extraction method: principal component analysis. Bold values indicate factor loadings with the highest correlation to the extracted component.

^a^Rotation method: Varimax with Kaiser normalization, rotation converged in three iterations.

**FIGURE 1:**
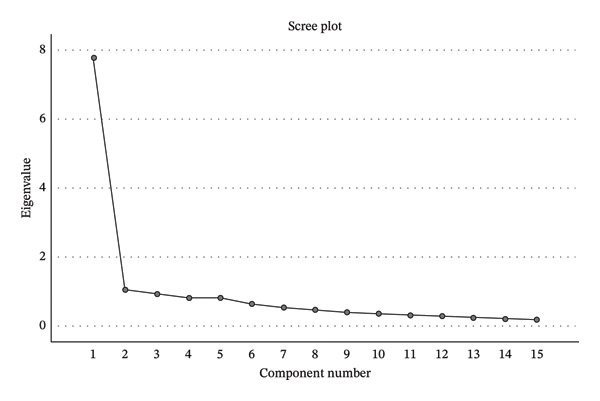
The selection of the number of components.

The intrinsic and extrinsic factors are defined empirically, not using the literature. In the study, we used questionnaire from the Institute of Public Health of Serbia ‘Dr Milan Jovanović Batut’, Belgrade, Serbia, which is a standard questionnaire for healthcare professionals’ satisfaction. Using the factor analysis, we have extracted two components from these items, which have 59% of cumulative variance explanation (Figure 1). These items are grouped in two components (dimensions), one hospital‐related and the other one healthcare professional–related. The components were saved as variables and correlated with questions regarding overall job and employee satisfaction, which is not part of the questionnaire. Further analysis showed that from such data, two factors can be extracted for a given condition (eigenvalue => 1). In fact, two factors explain 59% of the total variability of the data set, which is completely sufficient in this situation. In addition, Varimax rotation was performed. It was found that both factors similarly contribute to the cumulative explained variability, much more than non‐rotated data (Table 6).

If we had three components instead of two, the explained variability would be only 6% higher.

The analysis of the correlation of the obtained factors with individual variables regarding the explanation of how much each variable participates in the mentioned factors in the Varimax‐rotated solution is shown in Table 6. The first component included all variables whose coefficients are shown in bold font. The variables ‘relationship with the patient’ and ‘financial compensation for work’ were marginal and could belong to both factor 1 and factor 2 and therefore were arranged logically. Component 1 included extrinsically motivated factors, and component 2 intrinsically motivated job satisfaction factors.

The final correlation of the cumulative assessment of job satisfaction and two scores including extrinsically and intrinsically motivated factors of job satisfaction is shown in Table 7. Both intrinsic and extrinsic factors were significantly correlated with the overall job satisfaction. A somewhat higher correlation was seen between extrinsic factors and overall job satisfaction.

**TABLE 7:** Final correlation of the cumulative assessment of job satisfaction and two scores including extrinsically and intrinsically motivated factors of job satisfaction (*n* = 385).

		Extrinsic score	Intrinsic score
Overall job satisfaction	Correlation coefficient	0.567	0.526
	Sig. (2‐tailed)	0.000	0.000
	n	280	280

^∗∗^Correlation is significant at the 0.01 level (2‐tailed). Rho, Spearman’s rank correlation coefficient or Spearman’s *ρ*; *p*,  *p* value.

## 4. Discussion

### 4.1. Summary of Main Findings

The strongest factor of the overall job satisfaction among all employees was the appreciation and evaluation of work. For different professional groups, the key determinants were management and work organization, appreciation and evaluation of work, and the relationship with patients. Extrinsic factors had a greater impact on the overall job satisfaction.

### 4.2. Comparison With Previous Studies

In accordance with previous data [12, 16], only 15% of employees were satisfied with salaries, yet marking an increase in Serbia from 12.2% in 2013 [17]. In line with others [18, 19], 38.9% of participants were satisfied with job autonomy. Higher satisfaction with the work equipment (41.5%) compared to other institutions in Serbia (31%) in the past [17, 20] reflects state efforts in the past 8 years to improve healthcare equipment. As an indicator of high‐quality healthcare management [21], work equipment satisfaction suggests strong management in the two clinics. Only 37.7% of participants were satisfied with the time available to perform tasks, compared to 43.1% in a non‐tertiary centre [17]. Workload and high pressure in tertiary centres reduce overall job satisfaction [12, 16]. Other professionals in tertiary hospitals (geneticists, embryologists, psychologists, technical and administrative workers), also involved in the functioning of the hospitals and process of patient care, perceive a lower status compared to the medical professions, which decreases job satisfaction [22–24]. Overall job satisfaction correlated with work appreciation, clarity of instructions for administrative workers, and with time and skill utilization for technical workers, consistent to published data [24]. In contrast to data from Belgrade [17] and worldwide [22–24], technical and administrative workers expressed high levels of job satisfaction and satisfaction regarding the possibility to work using all knowledge, skills and abilities. This suggests good management in the two hospitals. Good management skills are predictors of job satisfaction in non‐medical personnel [22–24]. Possibility to work using all knowledge, abilities and skills is also an indicator of good management in medical institutions in general [25] and in tertiary centres [26]. Reported satisfaction with clinical leadership indicators is improved since earlier reports from Belgrade [17, 21]. Discontent with professional development is consistent with a previous Serbian study [17] but contrasting with global findings [27, 28]. High satisfaction with colleague cooperation suggests leadership by example and clear instruction [21].

Workplace stress negatively correlated and other factors positively correlated with overall job satisfaction. Such negative correlation was previously seen [29–33], as well as positive correlations [3, 12, 20, 27, 28]. Despite heavy workloads, patient complexity and high expectations in tertiary hospitals [8, 9], lower workplace stress (77.9%) compared to all (89.1%) Belgrade’s hospitals [17] may reflect effective management and organized workflows [30].

Extrinsic factors of job satisfaction influenced the overall job satisfaction more significantly than intrinsic, opposing to studies among nurses [34] and physicians [35, 36]. Evaluation of nurses and medical technicians by Minnesota Satisfaction Questionary (MSQ) revealed that 48% of participants were satisfied with intrinsic factors, while 22% of participants were satisfied with extrinsic factors of job satisfaction [34]. Similar results, although with lower overall job satisfaction, were reported in a study of nurses and medical technicians in Ethiopia [28]. These differences reflect the contexts of the studies: the first is conducted in a high‐income country, the second in a low‐income country and our study in a middle‐income country. In evaluation of relationship between intrinsic and extrinsic factors and overall job satisfaction, sense of calling and relationship with patients were analysed as intrinsic, and working hours, job autonomy and salary were evaluated as extrinsic factors of job satisfaction [36]. Higher sense of calling and longer working hours were associated with less job dissatisfaction. USA survey examined intrinsic and extrinsic factors in relation to career satisfaction, life satisfaction and clinical commitment [35]. Sense of calling was linked to life meaning and patient‐care commitment, while long‐term patient relationships correlated positively with career and life satisfaction. Our results indicate a slightly higher correlation between extrinsically motivated job satisfaction factor and overall job satisfaction. Previously cited studies indicate that intrinsic factors are important in the determination of job satisfaction and other aspects of well‐being of employees. While intrinsic motivators are associated with each measure of employee well‐being (satisfaction, meaning and commitment), extrinsic motivators are not associated with meaning or commitment [35]. A two‐stage cluster sampling study revealed that 60.8% of nurses were satisfied with job despite low salaries [28]. Understanding the effects of intrinsic factors helps in supporting employees’ well‐being. Workplace that supports intrinsic motivation increases job satisfaction and improves level of healthcare. Our data are along with data obtained with MSQ apropos intrinsic (sense of achievement, autonomy, opportunities to utilize knowledge, skills and personal growth), extrinsic factors (salary, working conditions and management) and overall job satisfaction [37–39]. Extrinsic factors (salary and working conditions) shape overall job satisfaction, aligning with literature [37]. Yet, a Romanian study reported a strong intrinsic dimension of job satisfaction, with extrinsic factors being less important [40]. The extrinsic factors predominated in our study, reflecting the high‐pressure, tertiary‐level context of the study, contradicting the Romanian study. Differences in the rank of specific satisfaction domains result from variances in measurement tools, study populations and clinical contexts. Addressing these aspects extended previous research by providing matching insights, not fully captured by MSQ‐based studies.

### 4.3. Strengths and Limitations of the Study

Few studies have identified intrinsic motivators in healthcare and examined their effects on professional commitment and well‐being of employees. Professional attitude of both physicians and nurses affects medical service quality, but studies focused on either nurses [28, 34] or physicians [35, 36]. Our study is the first one focused on all healthcare professionals in demanding workplace of tertiary obstetrics and gynaecology hospitals. We also designed a score to confidently evaluate the impact of intrinsic and extrinsic factors on overall job satisfaction. The homogeneous study population is a strength, reducing variability from differing professional roles and clinical contexts.

Our analysis examined the impact of intrinsic motivation on overall job satisfaction, putting the variables and their relations in a static form, hindering evaluation of casual relationships between variables. Lack of managerial position evaluation as a potential confounding factor is also limitation since the managerial position is a strong predictor of overall job satisfaction [41]. Our population largely consists of nurses in specific clinical setting. This implies bias and limits generalizability. Results do not represent nurses from other specialities or healthcare staff in different environments. Potential confounders (work‐related stress, shift patterns, institutional policies specific to obstetrics and gynaecology) influence the outcomes. While informative, results should be carefully taken due to outlined limitations. Future studies in diverse healthcare settings and professional groups may extend the generalizability of our results.

### 4.4. Further Research Directions

Improving work recognition, organizational support and workload management enhance staff engagement and improve patient experience. Distinguishing intrinsic and extrinsic factors of job satisfaction in demanding settings is vital. We can use our empirically supported framework for future studies in different professional group and healthcare context for evolution of more targeted measurement tools and intervention models. Investment in non‐financial incentives and transparent performance evaluation improves staff morale and service quality without big financial reform.

## 5. Conclusions

Factors influencing overall job satisfaction the most are work appreciation and evaluation, work management and organization, and relationship with the patient. Extrinsic factors correlate fairly higher with overall job satisfaction in middle‐income countries such as Serbia. Intrinsic factors correlate with overall job satisfaction too. Resource constraints do not justify neglecting intrinsic factors, given their minimal cost.

## Author Contributions

Conceptualization, A.R. and M.J.; methodology, M.P., I.S. and S.M‐R.; software, I.S. and B.S.; validation, I.S., B.S. and S.M‐R.; formal analysis, I.S. and B.S.; investigation, M.J., M.P., F.Dj. and V.J.; resources, F.Dj, V.J. and A.R.; data curation, I.S. and B.S.; writing original draft preparation, M.J., M.P. and A.R.; writing review and editing, S.M‐R., F.Dj. and M.P.; visualization, I.S. and B.S.; supervision, M.P., F.Dj. and V.J.; project administration, M.P, F.Dj. and A.R.

## Funding

This study was supported by the Ministry of Education, Science and Technological Development of the Republic of Serbia (Ministarstvo Prosvete, Nauke i Tehnološkog Razvoja RS, project No 200110).

## Disclosure

The funders had no role in the design of the study; in the collection, analyses, or interpretation of data; in the writing of the manuscript, or in the decision to publish the results. All authors have read and agreed to the published version of the manuscript.

## Ethics Statement

The study received ethical approval from the Ethics Committee of the Institute of Public Health of Serbia ‘Dr Milan Jovanović Batut’, with the approval number 2271/1.

## Conflicts of Interest

The authors declare no conflicts of interest.

## Data Availability

Data used in this study are available based on a reasonable request to the corresponding author.

## Appendix A National Survey of Employee Satisfaction

To what extent are you satisfied with:

Response scale: (1—Very dissatisfied, 2—Dissatisfied, 3—Neutral, 4—Satisfied, 5—Very satisfied, or Does not apply to me).

1. Adequacy of work equipment
2. Available time to perform your tasks
3. Available time to work with patients
4. Autonomy in performing your work—ability to make decisions
5. Opportunities to use all your knowledge, abilities and skills in your work
6. Recognition and evaluation of your work
7. Direct collaboration with colleagues
8. Direct collaboration with supervisors
9. Patients’ attitude towards you
10. Opportunities for professional development/continuing education
11. Financial compensation for your work
12. Leadership and organization of work in the institution
13. Receiving clear instructions regarding your job responsibilities
14. Opportunity to share your ideas with supervisors
15. How often do you feel tense, stressed or under pressure while performing your work? (1—Not at all, 2—A little, 3—Moderately, 4—A lot, 5—Very much)
16. Comparing your job satisfaction now to 5 years ago, are you: (1—More satisfied, 2—No difference, 3—Less satisfied)
17. Thinking about your work in the next 5 years, do you plan to: (1—Stay in the public health sector, 2—Move to the private health sector, 3—Work in a field outside healthcare, 4—Work abroad, 5—Not consider changing jobs)
18. Taking everything into account, how would you rate your overall satisfaction with your current job? (1—Very dissatisfied, 2—Dissatisfied, 3—Neither satisfied nor dissatisfied, 4—Satisfied, 5—Very satisfied)

General information.

19. Gender: (1—Male, 2—Female)
20. Age: (1—Under 35, 2—35–54, 3—Over 55)
21. Occupation: (1—Physician, 2—Nurse/Technician, 3—Healthcare staff—other, 4—Healthcare associate, 5—Administrative staff, 6—Technical staff)
22. Are you currently holding any managerial position? (1—Yes, 2—No)
23. Besides your current job at this institution, do you also work: (more than one answer allowed) (1—In teaching, 2—In private practice, 3—In another sector, 4—I do not work additionally)
24. Please indicate your smoking status: (1—I have never smoked, 2—I used to smoke, but I no longer do, 3—I currently smoke, but not every day, 4—I smoke daily)
